# Residential relocation and obesity after a natural disaster: A natural experiment from the 2011 Japan Earthquake and Tsunami

**DOI:** 10.1038/s41598-018-36906-y

**Published:** 2019-01-23

**Authors:** H. Hikichi, J. Aida, K. Kondo, T. Tsuboya, I. Kawachi

**Affiliations:** 1 School of Public Health, The University of Hong Kong, Hong Kong, Republic of China; 2 Department of International and Community Oral Health, Tohoku University Graduate School of Dentistry, Sendai, Japan; 3 Center for Preventive Medical Sciences, Chiba University, Chiba, Japan; 4 Center for Gerontology and Social Science, National Center for Geriatrics and Gerontology, Obu, Japan; 5 Department of Social and Behavioral Sciences, Harvard T.H. Chan School of Public Health, Boston, USA

## Abstract

Natural disasters are often associated with forced residential relocation, thereby affected people experience a change of food environment that results in the increased body mass index. However, there are a few studies that examined whether a change in food environment caused risk of obesity after a natural disaster. To address this question, we leveraged a natural experiment of residential relocation in the aftermath of the 2011 Japan Earthquake and Tsunami. Our baseline data came from a nationwide cohort study of older community-dwelling adults conducted 7 months prior to the disaster. By chance, one of the field sites (Iwanuma City, Miyagi Prefecture) was directly in the line of the tsunami. Approximately 2.5 years after the disaster, we ascertained the residential addresses and health status of 3,594 survivors aged 65 years or older (82.1% follow-up rate). Fixed effects multinomial logistic regression showed that shortened distances to food outlets/bars increased the risks of transitioning from BMI in the normal range (18.5–22.9) to obesity (≥25.0) (Odds ratios: 1.46 for supermarkets; 1.43 for bars; 1.44 times for fast food outlets). Radically changed food access after a natural disaster may raise the risk of obesity among older survivors.

## Introduction

Natural disasters are increasing in frequency and severity worldwide^1^. Due to population aging, older individuals are increasingly and disproportionately affected by disasters. In the aftermath of the 2011 Great East Japan Earthquake and Tsunami, which occurred on March 11, 2011, 89% of the post-disaster related deaths were older residents who were 65 years old or older^2^.

Experiences of natural disasters may be associated with weight gain. For example, incident depressive symptoms following disaster-related trauma might lead stress-related over-eating as a coping response. A panel survey in affected three municipalities of the 2011 Great East Japan Earthquake and Tsunami showed that residential relocation due to housing destruction was associated with increased body weight^3^. Other studies have reported increased alcohol intake^4^ and lower vegetable/fruit intake^5^ among survivors of the 2011 earthquake. However, the weight gain was measured only after the disaster, i.e., the study lacked information on the pre-disaster body weight of survivors, so that it is not possible to draw a strong causal inference.

Natural disasters are often associated with property destruction and forced residential relocation, thereby affected people experience a change of food environment that is hypothesized to affect people’s nutritional habits and body mass index (BMI)^6–8^. However, there are a few studies for the association between change in food environment and changes in weight status before vs. after natural disasters. Asking about pre-disaster conditions after the disaster is obviously subject to recall bias.

**Figure 1:**
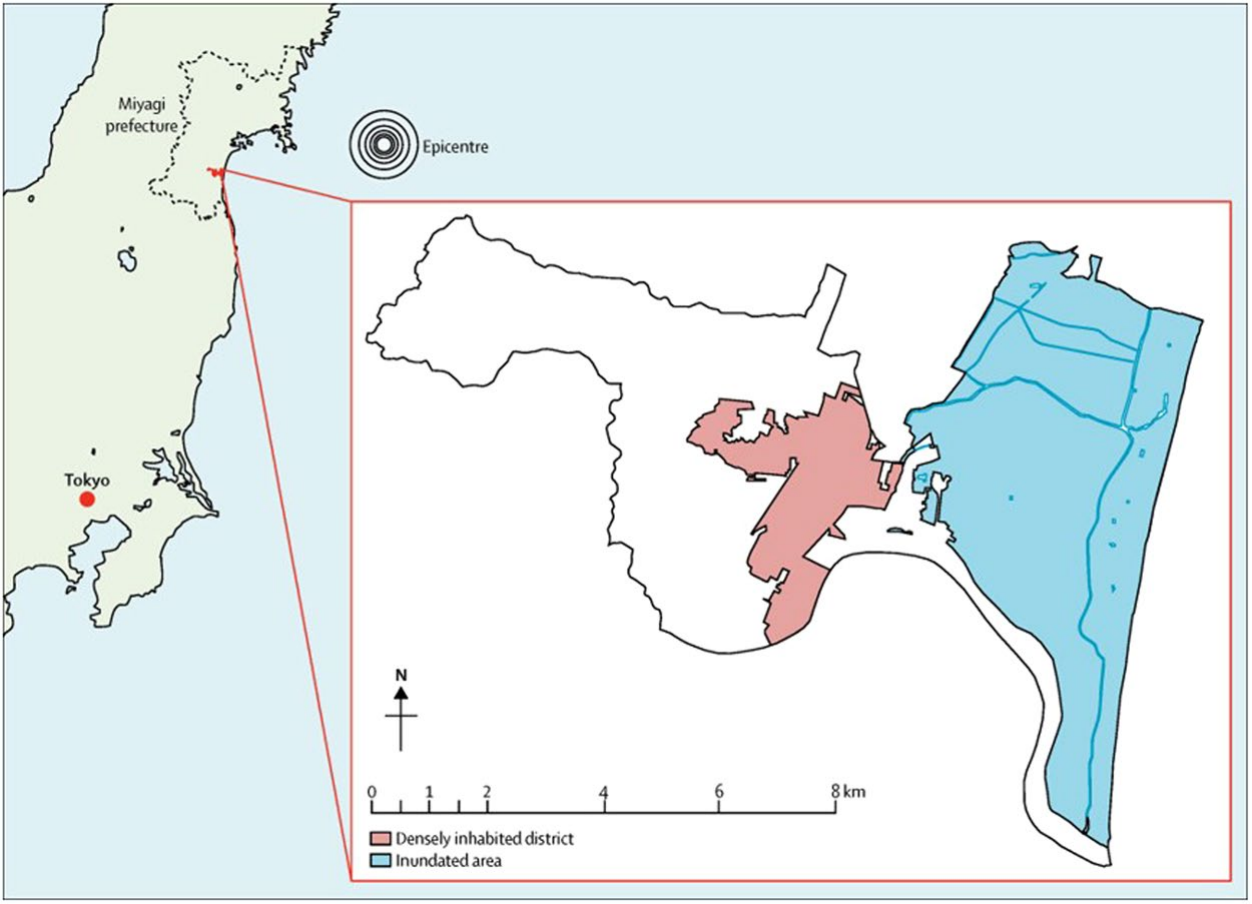
Map of inundated area in Iwanuma city, Japan. Reproduced from Hikichi, H. *et al*. Social capital and cognitive decline in the aftermath of a natural disaster: a natural experiment from the 2011 Great East Japan Earthquake and Tsunami. *The Lancet Planetary Health* 1 (3), e105-e113, DOI: 10.1016/S2542–5196(17)30041-4 (2017) (CC BY-NC-ND 4.0 license).

**Figure 2:**
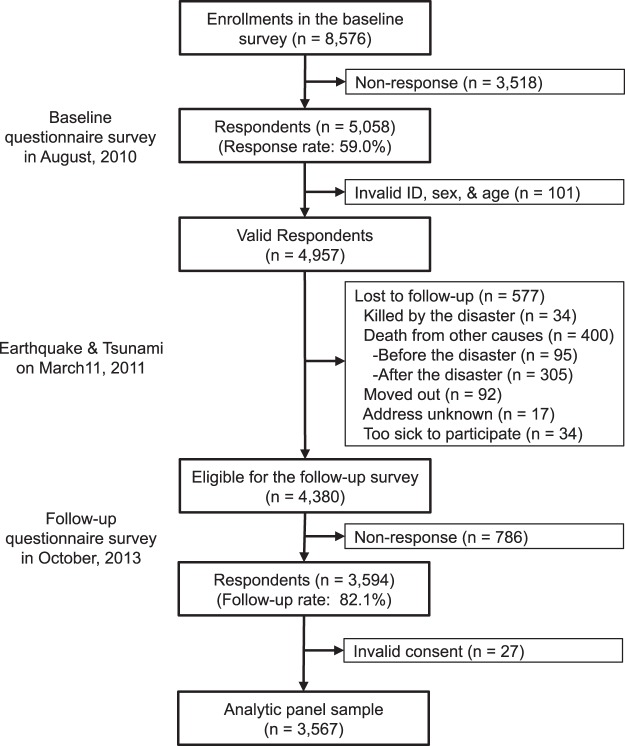
Participants flow in this study.

Here, we took advantage of a unique “natural experiment” stemming from the 2011 Great East Japan Earthquake and Tsunami, in which an estimated 18,500 people lost their lives^9^ and approximately 345,000 people were involuntarily displaced because of widespread sudden property destruction^10^. Our nationwide cohort study of aging, the Japan Gerontological Evaluation Study (JAGES), was established 7 months before the disaster to examine individual and community predictors of healthy aging. One of the field sites of the cohort was Iwanuma city in Miyagi Prefecture, located approximately 80-km west of the earthquake epicenter (Fig. 1). Approximately 2.5 years after the disaster, we recontacted 3,594 survivors to gather information about their disaster experiences and health status (82.1 percent follow-up rate) (Fig. 2).

**Figure 3:**
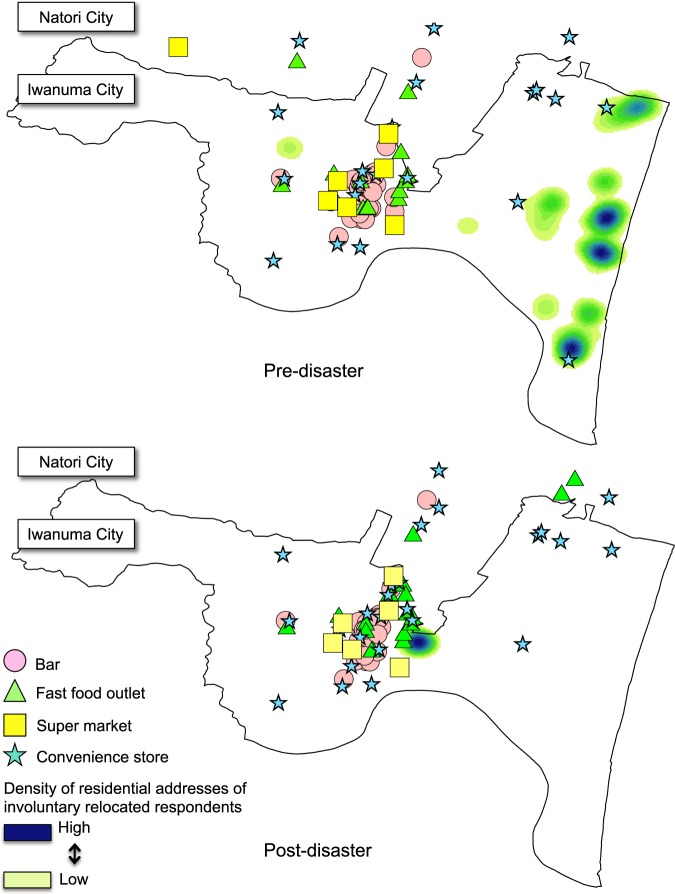
Density of displaced respondents’ addresses and locations of food outlets/bars.

Following the disaster, city officials directed the survivors who lost their homes to move into public prefabricated temporary housing villages [kasetsu jyutaku, resembling FEMA (Federal Emergency Management Agency)–style trailer housing communities in the United States] or to seek housing in the open rental market, both located in the central area of Iwanuma City. As a result (shown in Fig. 3), involuntarily relocated people experienced the greater convenience of access to local food establishments, including fast food outlets and bars. We hypothesized that shortened distances to food outlets/bar (supermarkets, fast food outlets, bars, and convenience stores) due to involuntary relocation would be associated with increased risk of weight gain.

## Methods

### Study participants

The Japan Gerontological Evaluation Study (JAGES) is a nationwide cohort study established in 2010 to examine prospectively the predictors of healthy aging. One of the field sites of the JAGES cohort is based in the city of Iwanuma (total population 44,187 in 2010)^11^ in Miyagi Prefecture. We conducted a census of all residents aged 65 years or older in August 2010 (n = 8,576), using the official residential register. The survey inquired about personal characteristics as well as their health status. The response rate to our baseline survey was 59.0% (n = 5,058), which is comparable to other surveys of community-dwelling residents.

The earthquake and tsunami occurred on March 11, 2011, seven months after the baseline survey. Iwanuma city is a coastal municipality located approximately 80 kilometers west of the earthquake epicenter, so that it was in the direct line of the tsunami that killed 180 residents, damaged 5,542 houses and inundated 48% of the land area (Fig. 1)^12^.

Approximately 2.5 years after the disaster (starting in October 2013), we conducted a follow-up survey of all survivors. The survey gathered information about personal experiences of disaster as well as updating their health status. Informed consent was obtained at the time of survey collection.

The detailed flow-chart of the analytic sample is presented in Fig. 2. Of the 4,380 eligible participants from the baseline survey, we managed to re-contact 3,594 individuals (follow-up rate: 82.1%). Our analytic sample comprises 3,567 individuals, after excluding respondents (n = 27) who returned invalid informed consent forms (e.g., signed by the next of kin rather than the individual).

### Outcome variable

Our primary outcome was BMI calculated from self-reported height and weight in both waves (2010 and 2013). The accuracy of self-reported BMI has been previously demonstrated in a Japanese older population, by comparing with physical measurements of BMI^13^. We categorized BMI into four categories, according to World Health Organization classification for Asian populations: <18.5 (underweight), 18.5–22.9 (normal weight), 23.0–24.9 (overweight), and ≥25.0 (obese)^14^.

### Explanatory variables

Our primary exposure variable was a change in distance (in kilometers) from each respondent’s residential address to the nearest food outlet or bar during the follow-up period. Data of fast food outlets (hamburger, fried chicken, pizza, pancake, noodle, and beef bowl) and bars were obtained from the corporate telephone directories in April 2010 and 2013 provided by Nippon Software Knowledge Corporation. The list of supermarkets (except for small grocery stores) and convenience stores (e.g., Seven-Eleven) were obtained from the Zenrin Geo Intelligence Company Limited, which cover the months from September 2009 to December 2010 (representing the pre-disaster period) and from March 2013 to May 2014 (representing the post-disaster period). Using ArcGIS Desktop version 10.4 (Esri, Redlands, California, USA), we then calculated the road network distance of each resident to the nearest bar, fast food outlet, supermarket, or convenience store^15^ in Iwanuma as well as in Natori city which the closest neighboring city and geographically accessible from Iwanuma (Fig. 3).

### Covariates

We selected time-varying demographic variables: age^16^, equivalized income^17^, spousal loss or divorce^18^, employment status^19^, and living alone^20^. Other time-invariant characteristics, such as sex and educational attainment, were omitted from our fixed effects regressions, because it was statistically differenced out in the model^21^. We also controlled for experiences of loss of relatives and/or friends during the disaster^22^.

We additionally examined a set of variables as potential risk factors for being obese. These variables included: current alcohol drinking^23^, current smoking^24^, the frequency of eating vegetables or fruits during the past one month^25^, the frequency of eating meat or fish during the past one month^26^, incident depressive symptoms^27^, and decreased daily walking time^28^, and neighborhood physical activity environment (within a 1 kilometer buffer zone from the residence)^29^. We asked respondents about the number of parks or sidewalks suitable for exercise or walking, the number of unwalkable places due to bumps or slopes, and the number of roads or intersections posing a high risk for traffic accidents.

Household income was equalized by the square root of the number of household members and grouped as 2 million Japanese yen or more versus under 2 million Japanese yen. Depressive symptoms were categorized into lower risk (4 points and lower) versus higher risk (5 points and higher)^30^.

### Statistical analysis

In the present study, we used a fixed effects multinomial logistic regression approach to examine the associations between changes in closest distance to a food outlet/bar and changes in BMI categories over time. This analysis estimates within-person probabilities of transition from the normal range at baseline to another range (underweight, overweight, or obese) at follow-up. The fixed effects approach effectively differences out all observed and unobserved time-invariant confounding factors (e.g., sex, educational attainment, and genetic factors)^31^. The reference category for our multinomial regression was individuals whose baseline BMI was within the normal range (18.5–22.9 kg/m2).

As a sensitivity analysis, the fixed effects linear regression using BMI as a continuous outcome was also conducted to check the linearity of the association between changes in distances to nearest food outlets and changes in BMI for the overall analytic sample regardless of respondents’ baseline BMI.

Because food outlets tend to be spatially clustered, it is difficult to isolate the effect of any specific type of outlet over another. Hence, we conducted an additional robustness check to examine whether a change in the exposure to any outlet within a 1 km buffer zone was associated with change in BMI. We measured the number (i.e. density) of four types of food outlets/bar within a 1 km radius of each residential address^8^ at both surveys. Before the disaster, approximately half of displaced respondents lived in places where there was no any food shop/restaurant within a 1 km radius of their residences.

To address potential bias due to missing data, we used multiple imputation by Markov Chain Monte Carlo method assuming missingness at random for explanatory variables and covariates. We created fifty imputed data sets and combined each result of analysis using the Stata command “mi estimate”. All analyses were performed using STATA version 14.0 (STATA Corp LP., College Station, Texas, USA).

### Ethics statement

The study was reviewed and approved by the Human Subjects Committee of the Harvard T. H. Chan School of Public Health, the Ethics Committee of the Tohoku University Graduate School of Medicine, the Research Ethics Committee of the Graduate School of Medicine, Chiba University, and the Research Ethics Committee involving Human Participants of the Nihon Fukushi University. Respondents signed on the informed consent form. We followed the STROBE Statement to report our observational study

## Results

We compared our analytic sample with the local (pre-disaster) census data for older residents (Table S1). The proportion of women is comparable to the actual census of older residents in Iwanuma city in October 2010 (56.5% for our sample, 57.2% for census data)^11^. The age distribution of our sample is also close to that of the local census data except for the group aged 85 years and over (6.2% and 13.2%, respectively)^11^. Our respondents were also somewhat more likely to be married (72.8%) compared to the census (64.7%)^32^. However, the proportion of employed individuals in our study (17.8%) is quite close to the census data (17.2%)^33^.

We also compared the characteristics of our analytic sample to non-respondents at the follow-up survey (Table S1). The sex distribution was similar, although our analytic sample was somewhat older than the non-respondents. The proportion of married people in our analytic sample (72.8%) was higher than among non-respondents (64.9%). More respondents were likely to be employed at the time of the follow-up survey (17.8%) compared with the non-respondents (14.0%). This could have resulted in some attrition bias, but the differences were not large.

Table 1 presents the characteristics of displaced respondents (n = 208) and non-displaced respondents (n = 3,359) at baseline (prior to the disaster) and at follow-up 2.5 years later. Our primary outcome was BMI calculated from self-reported height and weight in both waves (2010 and 2013), which were categorized into four categories according to World Health Organization classification for Asian populations: <18.5 (underweight), 18.5–22.9 (normal weight), 23.0–24.9 (overweight), and ≥25.0 (obese)^14^. The prevalence of obesity (BMI ≥ 25.0) was sharply increased among displaced respondents (25.0% to 35.1%), whereas non-displaced respondents reported a slightly decreased prevalence of obesity (26.9% to 26.6%).

**Table 1:** Characteristics of the analytic sample in baseline and follow-up survey.

	Displaced respondents (n = 208)				Non-displaced respondents (n = 3,359)			
	Baseline		Follow-up		Baseline		Follow-up	
	n	%	n	%	n	%	n	%
**BMI (WHO criterion for Asian)**								
Underweight (<18.5)	6	2.9	12	5.8	147	4.4	210	6.3
Normal range (18.5–22.9)	70	33.7	72	34.6	1274	37.9	1361	40.5
Over weight (23–24.9)	57	27.4	41	19.7	822	24.5	786	23.4
Obesity (≥25.0)	52	25.0	73	35.1	905	26.9	894	26.6
Missing	23	11.0	10	4.8	211	6.3	108	3.2
Total	208	100	208	100	3359	100	3359	100
**Closest distance to a supermarket**								
<0.5 km	6	2.8	21	10.1	624	18.6	624	18.6
>0.5 km –1.0 km	23	11.1	93	44.7	1475	43.9	1475	43.9
>1.0 km–2.0 km	5	2.4	82	39.4	424	12.6	424	12.6
>2.0 km	174	83.7	12	5.8	836	24.9	836	24.9
Missing	0	0.0	0	0.0	0	0.0	0	0.0
Total	208	100	208	100	3359	100	3359	100
**Closest distance to a bar**								
<0.5 km	23	11.0	53	25.5	1693	50.5	1613	48.1
>0.5 km –1.0 km	12	5.8	36	17.3	891	26.5	905	26.9
>1.0 km–2.0 km	6	2.9	110	52.9	166	4.9	208	6.2
>2.0 km	167	80.3	9	4.3	609	18.1	633	18.8
Missing	0	0.0	0	0.0	0	0.0	0	0.0
Total	208	100	208	100	3359	100	3359	100
**Closest distance to a fast food outlet**								
<0.5 km	11	5.2	52	25.0	1094	32.6	1275	38.0
>0.5 km –1.0 km	16	7.7	131	63.0	1177	35.0	954	28.3
>1.0 km–2.0 km	13	6.3	15	7.2	465	13.8	480	14.3
>2.0 km	168	80.8	10	4.8	623	18.6	650	19.4
Missing	0	0.0	0	0.0	0	0.0	0	0.0
Total	208	100	208	100	3359	100	3359	100
**Closest distance to a convenience store**								
<0.5 km	32	15.3	66	31.6	1114	33.1	1879	55.9
>0.5 km –1.0 km	59	28.4	28	13.5	1457	43.4	856	25.6
>1.0 km–2.0 km	33	15.9	112	53.9	595	17.7	448	13.3
>2.0 km	84	40.4	2	1.0	193	5.8	176	5.2
Missing	0	0.0	0	0.0	0	0.0	0	0.0
Total	208	100	208	100	3359	100	3359	100
**Loss of relatives and/or friends in the disaster** ^**a**^								
No			61	29.3			2106	62.7
Yes			142	68.3			1187	35.3
Missing			5	2.4			66	2.0
Total			208	100			3359	100
Age								
65–74 year	121	58.2	89	42.8	2006	59.7	1409	42.0
75– year	87	41.8	119	57.2	1353	40.3	1950	58.0
Missing	0	0.0	0	0.0	0	0.0	0	0.0
Total	208	100	208	100	3359	100	3359	100
**Equivalized income**								
<2.0 million JPY	105	50.5	106	51.0	1317	39.2	1480	44.0
≥2.0 million JPY	55	26.4	47	22.6	1434	42.7	1353	40.3
Missing	48	23.1	55	26.4	608	18.1	526	15.7
Total	208	100	208	100	3359	100	3359	100
**Bereavement or divorce**								
No	128	61.6	119	57.2	2332	69.4	2230	66.3
Yes	56	26.9	79	38.0	864	25.7	1013	30.2
Missing	24	11.5	10	4.8	163	4.9	116	3.5
Total	208	100	208	100	3359	100	3359	100
**Employment Status**								
Not working	129	62.0	168	80.8	2450	72.9	2837	84.5
Working	36	17.3	26	12.5	524	15.6	420	12.5
Missing	43	20.7	14	6.7	385	11.5	102	3.0
Total	208	100	208	100	3359	100	3359	100
**Living alone**								
No	181	87.0	174	83.7	404	12.0	449	13.4
Yes	15	7.2	25	12.0	475	14.2	512	15.2
Missing	12	5.8	9	4.3	156	4.6	47	1.4
Total	208	100	208	100	3359	100	3359	100
**Current drinking**								
No	139	66.8	149	71.6	2070	61.6	2273	67.7
Yes	56	26.9	56	26.9	1221	36.4	1065	31.7
Missing	13	6.3	3	1.5	68	2.0	21	0.6
Total	208	100	208	100	3359	100	3359	100
**Current smoking**								
No	146	70.2	179	86.1	2621	78.0	3087	91.9
Yes	36	17.3	26	12.5	467	13.9	252	7.5
Missing	26	12.5	3	1.4	271	8.1	20	0.6
Total	208	100	208	100	3359	100	3359	100
**Eating vegetable and fruit**								
Twice a day or more	108	51.9	76	36.5	1742	51.9	1574	46.9
Once a day	56	26.9	86	41.4	1034	30.8	1183	35.2
One to six times a week	36	17.3	37	17.8	531	15.8	558	16.6
Less than once a week	5	2.4	4	1.9	14	0.4	17	0.5
Missing	3	1.5	5	2.4	38	1.2	27	0.8
Total	208	100	208	100	3359	100	3359	100
**Eating meat and fish**								
Twice a day or more	14	6.7	17	8.2	385	11.5	416	12.4
Once a day	71	34.1	81	38.9	1233	36.7	1392	41.4
One to six times a week	106	51.0	99	47.6	1631	48.6	1480	44.1
Less than once a week	10	4.8	6	2.9	49	1.5	48	1.4
Missing	7	3.4	5	2.4	61	1.7	23	0.7
Total	208	100	208	100	3359	100	3359	100
**Depressive symptoms**								
Low risk	96	46.2	84	40.4	1994	59.4	1988	59.2
High risk	77	37.0	86	41.4	907	27.0	941	28.0
Missing	35	16.8	38	18.2	458	13.6	430	12.8
Total	208	100	208	100	3359	100	3359	100
**Walking time per day**								
90 minute and over	31	14.8	21	10.1	404	12.1	449	13.4
60 to 89 minute	18	8.7	22	10.6	475	14.1	512	15.2
30 to 59 minute	47	22.6	68	32.7	1136	33.8	1159	34.5
Under 30 minute	96	46.2	90	43.3	1188	35.4	1192	35.5
Missing	16	7.7	7	3.4	156	4.6	47	1.4
Total	208	100	208	100	3359	100	3359	100
**Parks or sidewalks suitable for exercise or walking in neighborhood**								
Many	26	12.5	27	12.9	187	5.6	211	6.3
Some	109	52.4	110	52.9	883	26.3	867	25.8
Few	48	23.1	35	16.8	1,822	54.2	1,842	54.8
None	11	5.3	8	3.9	291	8.7	245	7.3
Missing	14	6.7	28	13.5	176	5.2	194	5.8
Total	208	100	208	100	3,359	100	3,359	100
**Unwalkable environmental features in neighborhood: bumps or slopes**								
Many	5	2.3	3	1.4	387	11.5	450	13.4
Some	49	23.6	38	18.3	1,868	55.6	1,960	58.4
Few	112	53.9	108	51.9	826	24.6	720	21.4
None	26	12.5	27	13.0	108	3.2	85	2.5
Missing	16	7.7	32	15.4	170	5.1	144	4.3
Total	208	100	208	100	3,359	100	3,359	100
**Roads or intersections posing a high risk of traffic accidents in neighborhood**								
Many	24	11.5	12	5.7	32	1.0	87	2.5
Some	103	49.5	80	38.5	841	25.0	1,063	31.7
Few	62	29.8	78	37.5	1,894	56.4	1,759	52.4
None	7	3.4	8	3.9	421	12.5	301	9.0
Missing	12	5.8	30	14.4	171	5.1	149	4.4
Total	208	100	208	100	3,359	100	3,359	100
**Sex** ^**b**^								
Male	79	38.0			1,473	43.9		
Female	129	62.0			1,886	56.1		
Missing	0	0.0			0	0.0		
Total	208	100			3,359	100		
**Educational attainment** ^**c**^								
9 years and under	129	62.0			1,101	32.8		
10 years and over	67	32.2			2,132	63.4		
Missing	12	5.8			126	3.8		
Total	208	100			3,359	100		

^a^Empty cells at baseline due to unmeasured data before the disaster.

Abbreviations: JPY, Japanese Yen.

^b,c^Empty cells at baseline due to time-invariant variables.

Table 1 also shows that displaced survivors moved closer to most types of food outlets as a result of residential relocation. We calculated the road network distance of each residential address to the nearest bar, fast food outlet, supermarket, and convenience store, using the geographic information system. For example, 83.7% of displaced residents lived more than 2.0 km from the nearest supermarket before the disaster, but the proportion was reduced to 5.8% after relocation. By contrast, 24.9% of non-displaced residents lived >2.0 km from the nearest supermarket (which proportion remained unchanged, because they did not move). The proportion of displaced respondents who reported losing relatives and/or friends in the disaster was approximately two times higher than non-displaced respondents (68.3% and 35.3%, respectively).

In the present study, we used a fixed effects multinomial logistic regression approach to examine the associations between changes in the closest distance to a food outlet/bar and changes in BMI categories over time. This analysis estimates within-person probabilities of transition from the normal range (BMI 18.5–22.9 kg/m^2^) at baseline to another range (underweight (BMI < 18.5), overweight (23.0–24.9), or obese (BMI ≥ 25)) at follow-up^31^. Table 2 present the results of fixed effects multinomial logistic regression models correlating the change in distance (in km) to the closest supermarket, bar, fast food outlet, and convenience store and the odds ratios of transitioning to obesity, overweight, and underweight. The results showed that moving 1.0 kilometer closer to a supermarket, bar, or fast food outlet increased the odds of transitioning from BMI in the normal range (18.5–22.9) to obesity (≥25.0) (Odds ratio [OR] 1.46, 95% confidence interval [CI] 1.15, 1.86; OR 1.43, 95% CI 1.11, 1.86; OR 1.44, 95% CI 1.12, 1.86; respectively). Among the potential risk factor for obesity, drinking alcohol was also significantly associated with transitioning from normal range to obesity (ORs 1.17 to 1.24 through models with each food outlet/bar). Loss of loved ones and incident depressive symptoms were not significant for the risk of being obese after the disaster.

**Table 2:** Associations of a change in the closest distance to a food outlet/bar and a risk of transitioning from BMI in the normal range to obesity.

	BMI < 18.5^a^	BMI 23.0–24.9^a^	BMI ≥ 25.0^a^
	Adjusted odds (95% CI)^b^	Adjusted odds (95% CI)^b^	Adjusted odds (95% CI)^b^
**Per kilometer change in closest food outlets/bar**			
Supermarket	0.93 (0.59, 1.45)	1.07 (0.90, 1.28)	1.46 (1.15, 1.86)
Bar	0.91 (0.54, 1.51)	1.05 (0.87, 1.27)	1.43 (1.11, 1.86)
Fast food outlet	0.98 (0.61, 1.57)	1.01 (0.84, 1.20)	1.44 (1.12, 1.86)
Convenience store	0.79 (0.33, 1.86)	0.80 (0.51, 1.27)	1.10 (0.61, 1.98)

Abbreviations: BMI, body mass index; CI, confidence interval; km, kilometer.

^a^The odds ratio is risk of change in BMI from the normal range (18.5–22.9) at baseline to other ranges at follow-up.

^b^The model controlled for loss of relatives and/or friends, age, equivalent income, bereavement or divorce, employment, living alone, drinking, smoking, frequency eating vegetables and meats/fishes, depressive symptoms, decreased walking time, the number of parks or sidewalks suitable for exercise or walking, the number of unwalkable places due to bumps or slopes in neighborhood, and the number of roads or intersections posing a high risk of traffic accidents.

The results of fixed linear regression using continuous BMI as the outcome similarly indicated significant associations between distances and BMI (Table 3). A decrease in distance to the closest supermarket by 1.0 km resulted in an increment in continuous BMI by 0.08 units (95% CI 0.02, 0.15). Similarly, each kilometer decrease in distance to the nearest bar was associated with an increase in continuous BMI (0.08 units, 95% CI 0.01, 0.15, for the closest bar).

**Table 3:** Association of a change in the closest distance to a food outlet/bar and BMI (continuous variable).

	Super market	Bar	Fast food outlet	Convenience store
	Adjusted coef. (95% CI)	Adjusted coef. (95% CI)	Adjusted coef. (95% CI)	Adjusted coef. (95% CI)
Per kilometer change in closest food outlets/bar	0.08 (0.02, 0.15)	0.08 (0.01, 0.15)	0.05 (−0.02, 0.12)	0.13 (−0.04, 0.30)
Loss of relatives and/or friends	−0.02 (−0.13, 0.09)	−0.02 (−0.13, 0.10)	−0.01 (−0.13, 0.10)	−0.01 (−0.12, 0.10)
≥75 years old	−0.05 (−−0.21, 0.12)	−0.05 (−0.21, 0.12)	−0.05 (−0.21, 0.12)	−0.05 (−0.22, 0.11)
≥2 M equivalized income	−0.11 (−0.27, 0.05)	−0.11 (−0.27, 0.05)	−0.11 (−0.27, 0.05)	−0.12 (−0.28, 0.04)
Bereavement or divorce	−0.44 (−0.74, −0.15)	−0.44 (−0.73, −0.14)	−0.44 (−0.74, −0.15)	−0.44 (−0.74, −0.15)
Employment	0.12 (−0.03, 0.27)	0.12 (−0.03, 0.26)	0.12 (−0.03, 0.26)	0.12 (−0.03, 0.26)
Living alone	0.17 (−0.19, 0.52)	0.16 (−0.19, 0.52)	0.17 (−0.19, 0.52)	0.17 (−0.19, 0.52)
Drinking	0.55 (0.34, 0.76)	0.55 (0.34, 0.76)	0.55 (0.34, 0.77)	0.56 (0.34, 0.77)
Smoking	−0.22 (−0.51, 0.07)	−0.22 (−0.51, 0.07)	−0.22 (−0.51, 0.07)	−0.21 (−0.50, 0.08)
Frequency eating veg. & fruit	−0.06 (−0.14, 0.02)	−0.06 (−0.14, 0.02)	−0.06 (−0.14, 0.02)	−0.06 (−0.14, 0.02)
Frequency eating meat & fish	0.04 (−0.06, 0.14)	0.04 (−0.06, 0.14)	0.04 (−0.06, 0.14)	0.04 (−0.06, 0.14)
Depressive symptoms	−0.04 (−0.20, 0.12)	−0.04 (−0.20, 0.13)	−0.03 (−0.19, 0.13)	−0.03 (−0.19, 0.13)
Decreased walking time	−0.06 (−0.12, 0.01)	−0.06 (−0.12, 0.01)	−0.06 (−0.12, 0.01)	−0.06 (−0.12, 0.01)
Parks or sidewalks	−0.02 (−0.09, 0.06)	−0.02 (−0.09, 0.06)	−0.02 (−0.09, 0.07)	−0.02 (−0.09, 0.07)
Unwalkable places	0.06 (−0.04, 0.15)	0.06 (−0.04, 0.15)	0.06 (−0.04, 0.15)	0.06 (−0.03, 0.15)
Roads or intersections with high risk of traffic accidents	0.04 (−0.06, 0.15)	0.04 (−0.06, 0.15)	0.04 (−0.06, 0.15)	0.05 (−0.06, 0.15)

Abbreviations: BMI, body mass index; coef, coefficient; CI, confidence interval; km, kilometer; M, million; JPY, Japanese Yen; Veg, vegetable.

As shown in Table S2, an increase in the exposure to any food outlet/bar from 0 to ≥1 after the disaster was associated with an increment in continuous BMI by 0.70 units (95% CI 0.30, 1.11).

## Discussion

To our knowledge, this is the first study to demonstrate that involuntary relocation after a natural disaster is associated with a change in BMI among older survivors. The associations remained after statistically controlling for unobserved time-invariant variables as well as time-varying risk factors for change in BMI. Although shortened distance to the closest convenience store was not statistically significantly associated with risk of increasing BMI (Tables 2 and 3), the joint exposure to any type of food outlet/restaurants was statistically significant (Table S2). That is, improved food access may have caused an increase in BMI, regardless of the type of food outlet/restaurant.

There are several plausible mechanisms to explain weight gain among survivors of the disaster. For example, the stress and trauma experienced by survivors could lead to difficulties with sleep which is linked to increased BMI^34^. However, disaster-related trauma (i.e., loss of relatives and/or friends) and depressive symptoms were not significantly associated with increased BMI (Tables 2 and 3). Relocation to the trailer homes may have also resulted in diminished social interactions^35^, so that people had fewer opportunities to engage in physical activity, e.g. by walking every day to greet friends and neighbors. In our study, decreased daily walking time was also not significantly correlated with weight gain (Table 3). Thus, the remaining possible explanation for our findings is that improved access to restaurants, bars, and food retail outlets increased the opportunities for eating out and drinking, as changes in drinking habit are significantly associated with increased risk of becoming obese.

A major strength of this study is the availability of information pre-dating the disaster about BMI as well as other health conditions. Our design was, therefore, able to effectively address the problem of recall bias in most studies conducted in post-disaster settings.

A limitation is that selection bias might have arisen due to the 59% response rate to the baseline survey. However, this response rate is quite comparable to similar surveys involving community-dwelling residents^36^. In addition, we confirmed that the demographic profile of our participants is quite similar to the rest of Iwanuma residents aged 65 years or older (Table S1). Also, the response rate of our follow-up survey among survivors was quite high (82.1%). Owing to the compulsory residential registration system in Japan, only seventeen residents from the baseline sample could not be tracked (Fig. 2). Furthermore, BMI was self-reported and may include measurement error, although the accuracy of self-reports has been previously demonstrated in a Japanese older population^13^. Our findings may be still residually confounded by unobserved time-varying factors correlated with residential displacement. Our observations are also based on a relatively small sample of displaced survivors (n = 208), which may not be generalizable to displaced residents elsewhere. Nevertheless, our findings point to an important policy implication that planners should take into consideration access to services in making decisions about where to build temporary shelters in the aftermath of a disaster. In one sense, the local service access was “improved” for the disaster-affected residents in our study; but from the public health point of view, we also documented an unintended consequence, viz. dramatic weight gain.

## Supplementary information

Supplementary tables

## Data Availability

All data needed to evaluate the conclusions in the paper are present in the paper and/or the Supplementary Materials. The JAGES data used in this study will be made available upon request, as per NIH data access policies. The authors require the applicant to submit an analysis proposal to be reviewed by an internal JAGES committee to avoid duplication. Confidentiality concerns prevent us from depositing our data in a public repository. Authors requesting access to the Iwanuma data need to contact the principal investigator of the parent cohort (K.K.) and the Iwanuma sub-study principal investigator (I.K.) in writing. Proposals submitted by outside investigators will be discussed during the monthly investigators’ meeting to ensure that there is no overlap with ongoing analyses. If approval to access the data is granted, the JAGES researchers will request the outside investigator to help financially support our data manager’s time to prepare the data for outside use.
