# Supplementary material for: Residential relocation and obesity after a natural disaster: A natural experiment from the 2011 Japan Earthquake and Tsunami
Source: Sci Rep. 2019 Jan 23;9:374. doi: 10.1038/s41598-018-36906-y (PMC6344590; doi:10.1038/s41598-018-36906-y)
Supplement: Supplementary file 1 — Supplementary tables [file 41598_2018_36906_MOESM1_ESM.pdf]

Supplementary Materials

Residential relocation and obesity after a natural disaster: A natural experiment from the 2011 Japan Earthquake and Tsunami

H. Hikichi, J. Aida, K. Kondo, T. Tsuboya, and I. Kawachi

Correspondence to: hikichi@hku.hk

Tables S1: Comparison of characteristics among analytic sample at baseline, whole older population in 2010 local census, and non-respondents at follow-up survey

Table S2. Associations of change in density of food outlet/bar within a 1 km buffer zone and the risk of increased BMI

Table S1. Comparison of characteristics among analytic sample at baseline, whole older population in 2010 local census, and non-respondents at follow-up survey

	2010 all analytic sample		2010 local census		Non-respondents at follow-up survey	
	n [a]	%	n [a]	%	n [a]	%
Sex						
Male	1,552	43.5	3,735	42.8	326	41.5
Female	2,015	56.5	4,988	57.2	460	58.5
Age						
65-74 year	2,127	59.6	4,523	51.8	356	45.3
75-84 year	1,220	34.2	3,050	35.0	324	41.2
85 year and over	220	6.2	1,150	13.2	106	13.5
Marital status						
Married	2,460	72.8	5,618	64.7	469	64.9
Unmarried, widowed, & divorced	920	27.2	3,068	35.3	254	35.1
Employment status						
Working	560	17.8	1,493	17.2	93	14.0
Not working	2,579	82.2	7,169	82.8	573	86.0

[a] Marital status and employed status include missing values

Table S2. Associations of change in density of food outlet/bar within a 1 km buffer zone and the risk of increased BMI

	Adjusted coef. (95% CI)
Food outlet/bar within radious 1,000 m (ref: 0) [a]	0.70 (0.30, 1.11)
Loss of relatives and/or friends	-0.03 (-0.15, 0.09)
≥ 75 years old	-0.04 (-0.22, 0.13)
≥ 2 M equivalized income	-0.11 (-0.27, 0.06)
Bereavement or divorce	-0.38 (-0.71, -0.05)
Employment	0.12 (-0.04, 0.28)
Living alone	0.18 (-0.21, 0.56)
Drinking	0.54 (0.32, 0.77)
Smoking	-0.23 (-0.53, 0.06)
Frequency eating veg. & fruit	-0.05 (-0.14, 0.04)
Frequency eating meat & fish	0.03 (-0.06, 0.13)
Depressive symptoms	-0.02 (-0.18, 0.14)
Decreased walking time	-0.06 (-0.13, 0.01)
Parks or sidewalks	-0.03 (-0.12, 0.06)
Unwalkable places	0.10 (0.00, 0.19)
Roads or intersections with high risk of traffic accidents	0.04 (-0.07, 0.14)

Abbreviations: BMI, body mass index; coef, coefficient; CI, confidence interval; km, kilometer; M, million; JPY, Japanese Yen; Veg, vegetable

[a] Before the disaster, approximately half of the displaced respondents lived in location where there was no food shop/restaurant with a 1 kilometer radius of their residence.
